# Inherited Arrhythmias in Children

**Published:** 2008-05-01

**Authors:** Seshadri Balaji

**Affiliations:** Associate Professor, Department of Pediatrics, Division of Cardiology, Oregon Health & Science University, Portland OR USA

**Keywords:** Inherited arrhythmias, children

## Introduction

As our ability to discover new genetic mutations has increased, the list of inherited arrhythmias grows with each year. Virtually every form of arrhythmia has an inherited form, whether it is bradycardia, tachycardia or arrhythmic sudden death. This paper will therefore only focus mainly on those that are clinically most important in children.

## Bradycardia

### Sinus node disease

Sinus node dysfunction has been described as an inherited (autosomal recessive form) problem by Benson et al [1]. The cause seems to be certain specific mutations on the SCN5A gene. Patients with long QT type 3 (SCN5A mutation) have sinus bradycardia as part of the spectrum of abnormalities in this disease [2]. Atrial standstill is a rare condition where the atrium is silent i.e. there is no electrical activity present and also the atrium is not excitable by pacing. Rare, inherited forms of this condition have been described [3,4].

### AV node disease

The spectrum of AV block associated with ASD and sudden death has been described secondary to mutations in the NKX2.5 gene [5]. Some patients with Long QT type 3 and others with Brugada syndrome (both of which are due to mutations in the SCN5A gene) have associated complete heart block (CHB) [2-5]. Patients with myotonic dystrophy which is inherited in auto dominant fashion have CHB. This condition is also associated with cardiomyopathy and SCD [6].

## Tachycardia

Inherited forms of suprventricular tachycardia (SVT) including ectopic atrial tachycardia, AV nodal reentrant tachycardia, WPW syndrome, atrial fibrillation have all been described [7-11]. However, they are rare. Inherited ventricular tachycardia (VT) such as idiopathic right ventricular out flow tract left ventricular fascicular VT and arrhythmogenic right ventricular dysplasia (ARVD) have also been described [12,13]. ARVD will be elaborated under the sudden death section (see below).

## Sudden Death

There has been huge progress in the last few decades in our understanding of familial causes of sudden death (SD). The genetic basis of conditions like long QT syndrome, hypertrophic cardiomyopathy, Brugada syndrome, catecholaminergic polymorphic VT etc have been elucidated.

### Long QT syndrome

Since its first description in a Norwegian family by Jervell and Lange-Nielsen, long QT syndrome has been known to be familial [14]. The first reported kindred had associated deafness and was inherited as an autosomal recessive condition. Subsequently, independent reports by Romano and Ward described an autosomal dominant type of long QT syndrome [15,16]. Patients with long QT syndrome present with symptoms of syncope, near-miss sudden death or are mistakenly thought to have epilepsy. The key finding is an abnormally prolonged QT interval on the ECG. The QTc (using Bazet's formula) usually exceeds 460 ms. Syncope or sudden death may be precipitated by exercise or excitement (this predominates in LQT1), sudden fright especially from auditory stimuli (predominates in LQT2) or during sleep (dominates LQT3) [17,18]. Schwartz et al have proposed a scoring system to make the diagnosis in atypical cases [19].

Currently, 10 genetic subtypes of this condition have been described. The three most common forms account for about 80% of all described cases and the others have been noted mainly in rare families. The genes involved mainly code for either the cardiac potassium channel (Long QT1 and 2) or the sodium channel (long QT3). Genetic testing has become commercially available in the USA, although the gene mutation is identifiable in only ~60% of patients with proven long QT syndrome.

The treatment of long QT syndrome was revolutionized by the arrival of beta blockers. From the earliest report, it was clear that epinephrine was detrimental to the heart and could precipitate torsade-des-pointes or ventricular fibrillation. Beta blockers have been suggested to achieve a ten-fold reduction in the incidence of arrhythmias [20]. Other therapies shown to help are: pacing, left cervical sympathectomy, and in selected cases, mexiletene. The arrival of the automatic implantable cardioverter-defibrillator has given us the option of a safety net in patients who do not respond to these above treatments.

Infantile LQT is a serious disorder which presents with paroxysms of arrhythmia and bradycardia. Both sinus node disease and AV node block have been described. Additional, excessive prolongation of the QT interval creates a form of pseudo- AV block whereby every other P wave fails to capture the ventricle because the ventricle is refractory [21].

Recent evidence suggests that with the use of beta-blockers, a very high level of protection can be achieved in patients with LQT1 [22]. Therefore it is rare for such patients to need an ICD. Conversely, there is some evidence that drug therapy is not as protective in LQT3, and therefore, an ICD is more beneficial in this group of patients. LQT 2 seems to fall between 1 and 2 in terms of its risk and the potential need for an ICD.

### Short QT syndrome

More recently, there has been interest in the short QT syndrome where patients often have a QTc of <340 (usually <300 ms) [23]. So far, three genetic subtypes have been identified. Beta-blockers are not protective and may even be detrimental. There is evidence that the class Ia antiarrhythmics (quinidine) may be protective in some patients.

### Hypertrophic Cardiomyopathy

Hypertrophic Cardiomyopathy (HCM) is a complex disease with a varying phenotype, and the result of a multitude of mutations in genes encoding the cardiac contractile proteins (actin, myosin, troponin) [24,25]. Mostly inherited as an autosomal dominant problem, there is wide variability in penetrance, and in phenotypic expression. Patients with HCM can have a significant incidence of ventricular arrhythmias and SD. There is a rare subtype called Danon disease with HCM associated with WPW, which has a high incidence of arrhythmias as well [26].

Certain gene mutations have been identified which are associated with SD in HCM. In clinical practice, risk stratification for SD in HCM is a problem. McKenna and colleagues have proposed a set of criteria, which should alert the clinician to a high risk of SD [27]. These include: history of near-miss SD, family history of SD, history of syncope, demonstration of non-sustained VT by ECG monitoring, excessive thickening of the interventricular septum (>3 cm in adults), a flat BP profile with exercise. The presence of two of these risk factors should lead to intensive evaluation, and the presence of three should be an indication for an ICD.

### Arrhythmogenic RV Dysplasia

ARVD is characterized by fibro-fatty infiltration of the myocardium. While the RV is predominantly affected, it can also affect the LV. The infiltration leads to islands of myocardium separated by the fibro-fatty areas, and the potential for development of areas thinning, aneurysm/diverticulae formation, akinesia, and most importantly, the development of VT and VF [13].

The variable phenotype makes this a difficult diagnosis to prove. Ventriculography or MRI of the ventricles can help. Biopsy may help if positive, but since this is a spotty disease, can be missed by endomyocardial biopsy. Genetic research so far has identified mutations in the plakophilin gene as a possible cause of this disease. ARVD is an extremely rare problem in children because the phenotype often does not manifest itself until late adolescence.

### Brugada syndrome

Recently described, this syndrome is characterized by sudden death, especially in young (teenage/young adult) males, with the ECG finding of ST elevation in the anterior chest leads (V1 and V2). The ECG findings can be easily missed or can be intermittent, and therefore, special vigilance has been suggested to makes this diagnosis [28,29]. Brugada syndrome has been shown to be caused by certain mutations in the SCN5A gene (the same one responsible for LQT3). Sodium channel blockers (such as flecainide/propafenone etc) can be used to unmask the problem on the ECG [28]. Drug therapy has so far not been shown to be effective, and risk stratification is also fraught with problems. Presence of the Brugada pattern on ECG, symptoms such as syncope, and male sex are thought be indications for the implantation of an ICD [29].

### Catecholaminergic Polymorphic VT

Catecholaminergic Polymorphic VT (CPVT) is a condition characterized by exercise- or stress-induced ventricular tachyarrhythmias and syncope, or sudden death, usually in the pediatric age group. Familial occurrence is noted in about 30% of cases and inheritance is autosomal dominant or recessive, with high penetrance [30]. Genetic studies have implicated mutations in the cardiac ryanodine receptor gene (RyR2) in autosomal dominant pedigrees, and the calsequestrin gene (CASQ2) mutations seen in recessive cases. Additionally, Ankyrin-B mutations have also been implicated in this condition. CPVT carries a high mortality in untreated cases ranging from 30% to 50% before the age of 20-30 years. While beta-blocker therapy can help, the efficacy is low and symptoms have been shown to occur even with a single missed dose. Because of this, the trend is to implant a defibrillator.
